# Characterization of a negative transcriptional element in the *BRCA1 *promoter

**DOI:** 10.1186/bcr1753

**Published:** 2007-07-30

**Authors:** Gwen MacDonald, Melissa Stramwasser, Christopher R Mueller

**Affiliations:** 1 Queen's Cancer Research Institute, Queen's University, Kingston, Ontario, Canada, K7L 3N6; 2 Department of Biochemistry, Queen's University, Kingston, Ontario, Canada, K7L 3N6; 3 Department of Pathology and Molecular Medicine, Queen's University, Kingston, Ontario, Canada, K7L 3N6

## Abstract

**Introduction:**

Decreased transcription of the *BRCA1 *gene has previously been observed to occur in sporadic breast tumours, making elucidation of the mechanisms regulating the expression of this gene important for our understanding of the etiology of the disease.

**Methods:**

Transcriptional elements involved in the regulation of the *BRCA1 *promoter were analysed by co-transfection experiments into the human MCF-7 and T-47D breast cancer cell lines.

**Results:**

We have identified a repressor element, referred to as the UP site, within the proximal *BRCA1 *promoter whose inactivation results in increased promoter activity. An E2F recognition element, previously suggested to mediate repression via E2F-6, is adjacent to the UP site and its inactivation also leads to increased *BRCA1 *expression. These two elements appear to form a composite repressor element whose combined effect is additive. The UP element is composed of two sequences, one of which binds the ubiquitously expressed *ets *family transcription factor GABP alpha/beta. This site is distinct from a previously identified GABP alpha/beta site, the RIBS element, though the RIBS site appears to be necessary for derepression of the promoter via mutations in the UP site. Knockdown of GABP alpha using an shRNA vector confirms that this protein is important for the function of both the RIBS and UP sites.

**Conclusion:**

The identification of a repressor element in the *BRCA1 *promoter brings a new level of complexity to the regulation of *BRCA1 *expression. The elements characterized here may play a normal role in the integration of a variety of signals, including two different growth related pathways, and it is possible that loss of the ability to derepress the *BRCA1 *promoter during critical periods may contribute to breast transformation.

## Introduction

The *BRCA1 *tumour suppressor gene plays a central role in the development of breast cancer. In familial cancer, inheritance of a mutant allele leads to tumour formation through the loss of heterozygosity of this locus [1]. For other identified tumour suppressor genes, mutations are generally responsible for both the hereditary and sporadic forms of the same type of cancer. However, no consistent pattern of mutation of the *BRCA1 *gene has ever been identified in sporadic breast cancer tumours [2-4]. In contrast, the loss of *BRCA1 *expression appears to be an important mechanism driving tumour formation in sporadic breast cancer cases [5]. There is evidence to suggest that epigenetic changes and preferential methylation of sites within the *BRCA1 *promoter region can lead to this down-regulation of expression; however, collectively, these mechanisms are implicated in only a small percentage of sporadic tumours [6]. These data suggest that transcriptional regulation of the *BRCA1 *gene may play a major role in the loss of its expression.

As a protein involved in a variety of cellular processes, including repair, recombination and transcriptional regulation [7], the disregulation of BRCA1 activity is expected to have a wide variety of effects. Artificially increasing the expression of BRCA1 in tumour cell lines has been shown to decrease growth and induce apoptosis [5]. Selective inactivation of the *BRCA1 *gene in the breast results in breast hyperplasia, blunted ductal development and tumour formation [8]. Low BRCA1 levels in human breast cancers are correlated with tumour progression, increased malignancy and poor prognosis [9-11]. This suggests that altered BRCA1 levels have an ongoing effect on cellular processes.

The transcriptional regulation of *BRCA1 *expression is complex, being modulated by a variety of hormones, developmental cues and other effectors (reviewed in [12]). The *BRCA1 *gene is transcribed divergently with the *NBR2 *gene, with only several hundred base-pairs between them [13,14]. A minimal bidirectional promoter element has been defined and is located some 200 base-pairs upstream of the *BRCA1 *transcriptional start site [15]. Within this region we have previously identified a critical element, referred to as the RIBS site (EcoRI Band Shift), which interacts with the *ets *transcription factor GABP alpha/beta [16]. Functional analysis of the *BRCA1 *promoter revealed that the RIBS site is important for promoter activity, and appears to be differentially regulated in the MCF-7 and T-47D cell lines, with this element being less active in T-47D cells [16].

GABP alpha/beta is a ubiquitous transcription factor that binds to GA-rich sequences [17,18]. The human complex exists as a heterodimer consisting of an *ets *family helix-loop-helix DNA-binding domain subunit (GABP alpha), and a Notch-Ankyrin repeat family subunit (GABP beta) that contains the activation domain as well as a domain required for the formation of tetrameric complexes. GABP alpha/beta has been implicated in the regulation of genes in response to cell growth, activation of respiration related genes [19] and as a downstream mediator of ErbB3 and ErbB4 signalling [20]. The interaction of the GABP complex subunits with each other and with numerous other transcription factors and co-activators defines its ability to regulate target gene transcription.

Here, an element in the *BRCA1 *proximal promoter, referred to as the UP (UPstream) site, is identified and characterized. This site appears to act as a repressor, as mutation of key residues in this element results in an increase in the transcriptional activity of the promoter. Mutation of a downstream E2F site appears to have the same effect on promoter activity. The UP site is shown to contain a GABP alpha/beta binding element that is required for repressor activity. Both deletion constructs and experiments using a small hairpin RNA (shRNA) vector against the GABP alpha subunit confirm that the RIBS element and the GABP complex are required for activation of the promoter as a result of UP mutations.

## Materials and methods

### Methylation interference assay

**Figure 1:**
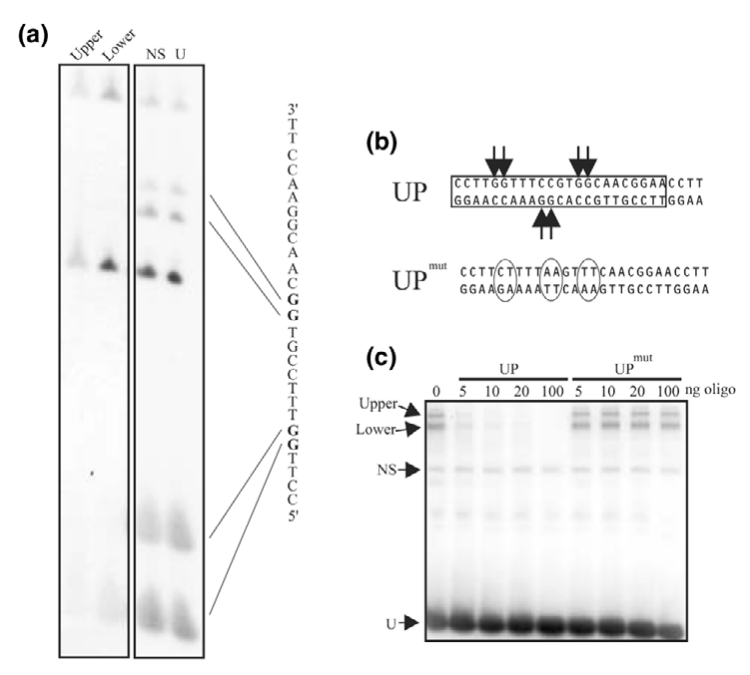
Methylation interference assay of the UP site. **(a) **Nuclear proteins were used in a bandshift assay with a UP probe that had been chemically methylated. The various complexes indicated in (c) were extracted, chemically cleaved, individually separated on a denaturing gel and autoradiographed. The G residues whose methylation blocks binding to the upper and lower complexes are shown in bold in the sequence of the UP site. Only the non-coding strand is shown. **(b) **Location of G residues sensitive to methylation. Methylation of six G residues, indicated by the arrows, block binding to the upper and lower complexes. A UP oligonucleotide (UP^mut^) with mutations at these residues (circled) was created. **(c) **Binding of nuclear proteins to wild-type and mutant UP probes. A bandshift assay was preformed with nuclear extracts using the wild-type UP oligonucleotide as a probe. The indicated amounts of cold wild-type UP or mutant UP oligonucleotides (oligo) were added into the reaction. The complexes correspond to Upper, Lower, non-specific (NS) and unbound (U).

This protocol was modified from Siebenlist and Gilbert [21]. One hundred nanograms of the individual strands of the UP oligonucleotide (sequence in Figure 1) were labelled using T4 polynucleotide kinase and gamma ^32^P-ATP. The reaction was heat inactivated and an excess of cold oligonucleotide was annealed to the labelled strand. The single-stranded ends were filled in using Klenow DNA polymerase. The labelled oligonucleotide was purified by exclusion chromatography on a Sephadex G-50 column in DMS buffer (50 mM Na-cacodylate pH 8.0, 1 mM EDTA, 50 mM NaCl). Methylation of the labelled oligonucleotide was carried out in a 200 μl reaction with 2 μg of poly dIdC and 1 μl of dimethyl sulphate at 37°C for 20 minutes. The reaction was terminated with 50 μl DMS stop buffer (1.5 M NaOAc pH 7.0, 100 μg/ml tRNA, 1.0 M beta-mercaptoethanol) with 10 μg of poly dIdC also added. The product was precipitated with ethanol and resuspended in TEN50. This probe was used in bandshift assays as described and both DNA:protein and free DNA was isolated from the wet gel using electroelution. The DNA was precipitated and resuspended in 90 μl of water. Piperidine (10 μl) was added and incubated at 90°C for 30 minutes. Piperidine was removed by lyophiliszation with several rounds of water addition. The fragmented DNA was then eletrophoresed on a 20% urea-polyacrylamide gel, dried and autoradiographed.

### Cloning

Creation of the L6-pGL2 and L6DR-pGL2 *BRCA1 *promoter constructs has been described previously [16]. For these experiments these promoter constructs were cut with the restriction enzymes *Sma*I and *Hin*dIII and re-cloned into the pRL reporter vector (Promega, Madison, WI, USA), which had been cut with *Xho*I, blunted using Klenow, and then cut with *Hin*dIII. Creation of the L6-mUP-pGL2 construct was achieved using nested mutagenic primers and L6-pGL2 as the template. The products of these reactions were gel purified, annealed and a third PCR reaction was then performed to amplify the full-length mutated L6 promoter. The insert was cloned into the pGL2 vector using the restriction enzymes *Nhe*I and *Hin*dIII, and then re-cloned into the pRL vector as described above. The L6-mE2F-pRL construct was created in a similar manner, using nested primers and the L6-pRL construct as the template, and the *Nhe*I and *Hin*dIII restriction sites. The L6DR-mUP-pRL and L6DR-mE2F-pRL constructs were created by cutting the L6-mUP-pRL and L6-mE2F-pRL constructs, respectively, with the restriction enzymes *Msc*I and *Hin*dIII. The mutant fragments were then cloned into the L6DR-pRL vector using these same sites. The RIBS multimer in the GF-TATA-luc vector has been described previously [16]. The UP multimer was cloned upstream of the TATA box in a similar manner.

The H1 vector primers were derived from [22] and used along with human genomic DNA to amplify the proximal promoter from the histone H1 gene. This fragment was cloned into pBS+ using *Eco*RI and *Hin*dIII. Oligonucleotides corresponding to sequences in GABP alpha and luciferase were synthesized and annealed and the resulting fragments were cloned into the H1-pBS+ vector using the *Bgl*II and *Hin*dIII restriction sites.

All restriction enzymes were obtained from NEB (Pickering, ON, Canada). Primer sequences are available from the authors upon request. All constructs were verified by sequencing.

### Production of recombinant proteins

PCR was used to amplify the coding regions of the human GABP alpha and GABP beta genes, which were the kind gift of J-I Sawada and H Hanada, using the cDNAs in pCAGGs as the templates and the primer pairs GABP alpha-(ATG) GGG TCT AGA ATG ACT AAA AGA GAA GC, GABP alpha-(TERM) GGG AAG CTT TCA ATT ATC CTT TTC CG and GABP beta-(ATG) GGG TCT AGA ATG TCC CTG GTA GAT TTG G, GABP beta-(TERM) GGG GTC GAC GTT CAT TTC AAT TAA ACA GC, respectively. The products were then cloned into the pMAL-C2 vector using *Xba*I/*Hin*dIII and *Xba*I/*Sal*I, respectively. The recombinant proteins were expressed and purified according to the manufacturer's protocols. The purified proteins were eluted with maltose in nuclear dialysis buffer (25 mM HEPES pH 7.6, 0.1 mM EDTA, 40 mM KCl, 10% glycerol, 1 mM DTT).

### Cell culture

The cell lines MCF-7 and T-47D were maintained in RPMI 1640 medium supplemented with 10% fetal bovine serum, 100 units/ml penicillin, and 100 g/ml streptomycin. HeLa cell lines were maintained in Dulbecco's modified Eagle's medium supplemented with 10% fetal bovine serum, 100 units/ml penicillin, and 100 μg/ml streptomycin. All lines were obtained from ATCC (AT CC, Manassas, VA, USA) and maintained at 37°C with 5% CO_2_.

### Transient transfections and luciferase assays

For all transfections for which luciferase activities were measured, cells were seeded in 12-well plates at a density of 1 × 10^5 ^cells/ml, 24 hours before transfection. All transfections were carried out using 0.75 μl per well FuGene6 transfection reagent (Roche Applied Science, Laval, QC, Canada), according to the manufacturer's instructions. To examine the relative activity of each promoter, 225 ng of each *BRCA1 *reporter construct was transfected along with 25 ng CMV-Luc internal control for a total of 250 ng DNA per well. For the overexpression studies, each condition consisted of 25 ng CMV-Luc internal control, 25 ng of each of the GABP expression vectors or their corresponding empty vector controls, and 175 ng of the specified renilla luciferase reporter vector, for a total of 250 ng of DNA per well. For the knock-down studies, 50 ng of the shRNA construct or its empty vector were used in place of the expression vectors. Each condition was performed in triplicate. The cells were lysed 48 hours post-transfection using passive lysis buffer, and assayed using the Dual-Luciferase Assay System (Promega) as per the manufacturer's instructions. In order to test the effectiveness of the shRNA constructs, HeLa cells were plated on 12-well plates at a density of 4 × 10^4 ^cells/ml, 24 hours prior to transfection. Transfections were performed using 3 μl of FuGene transfection reagent and 2 μg of shRNA plasmid, as per the manufacturer's instructions. Seventy-two hours post-transfection, the cells were scraped and lysed using 50 μl of modified RIPA buffer (50 mM Tris-HCL pH 7.4, 1% Igepal C630, 0.25% Na-deoxycholate, 150 mM NaCl, 1 mM EDTA, 1 mM Phenyl-Methyl-Sulfonyl-Floride, 1 μg/ml each of aprotinin, leupeptin and pepstatin, 1 mM Na3VO4, 1 mM NaF) for 15 minutes at 4°C. An equal amount of 2× SDS-PAGE loading buffer was added to each lysate.

### Western blotting

In order to detect GABP alpha, proteins were resolved by SDS-PAGE, blotted onto a nitrocellulose membrane and probed with an antibody directed against human GABP alpha. Secondary antibody detection was achieved by chemiluminescence (Pierce, Rockford, IL, USA). To confirm equal loading, the blots were then washed with PBS and re-probed with an antibody directed against Sp1 (Santa Cruz Biotechnology, #sc59 Santa Cruz, CA, USA). Secondary antibody detection was achieved as described above.

### Antibodies used for western blotting

Rabbit antibodies were prepared by Chemicon (Temecula, CA, USA), and were raised against a peptide (ASQEQQMNEIC) that corresponds to a region between the pointed and *ets *domain of human GABP alpha, which is conserved between mouse, rat and human sequences. A peptide (MQNQINTNPEC) corresponding to a region to the amino-terminal side of the ankyrin repeats and also conserved between mouse, rat and human was used to create antibodies against human GABP beta.

### Bandshift reactions

Bandshift conditions used were the same as outlined in [16]. Supershift assays were performed as described in [23], using the Santa Cruz antibodies GABP alpha (H-2 X), CREB-1 (C-21) and Ets2 (C-20).

### Oligonucleotides

Specific oligonucleotides used are as indicated in the Figures and the sequences are available on request.

### Chromatin immunoprecipitation assays

Chromatin immunoprecipitation (ChIP) assays were carried out with MCF-7 cells using the ChIP-It Express Enzymatic kit (Active Motif, Carlsbad, CA, USA) as per the manufacturer's instructions. Each reaction was performed using chromatin from 2 × 10^6 ^cells and 2 μg of affinity-purified antibody or 5 μl of whole sera. Affinity-purified antibodies used include: GABP alpha (Santa-Cruz, (H-180 X)), haemagglutinin (Santa-Cruz, (Y-11)), and acetylated-histone H3 (Upstate Biotechnology (Lake Placid, NY, USA). Whole serum antibodies used include GABP beta (Chemicon) and pre-immune serum (Chemicon).

## Results

### Identification of the UP binding site in the *BRCA1 *proximal promoter

Footprinting analysis of the *BRCA1 *promoter had identified an element, referred to here as the UP site, located near the transcriptional start site (data not shown). Bandshift analysis of a variety of nuclear extracts derived from human breast cancer cell lines indicated that two slowly migrating complexes were formed with a UP probe. Self competition experiments confirmed that this interaction was specific. In order to further characterize the interaction between the transcription factor complexes and the UP site, individual nucleotide contact points were identified using methylation interference assays with the UP oligonucleotide (Figure 1a). These results indicated that a series of G residues through the 5' end of the site were necessary for interaction with the protein complex. To characterize the specificity of the transcription factor-DNA complexes formed, gel shift assays were preformed. Mutation of these nucleotides in the context of a double-stranded oligonucleotide corresponding to the UP site (Figure 1b) eliminates specific binding of factors to this site (data not shown), while competition assays with the wild-type or mutant probes confirm that the mutant oligonucleotide no longer binds (Figure 1c).

### The UP site acts as a repressor

**Figure 2:**
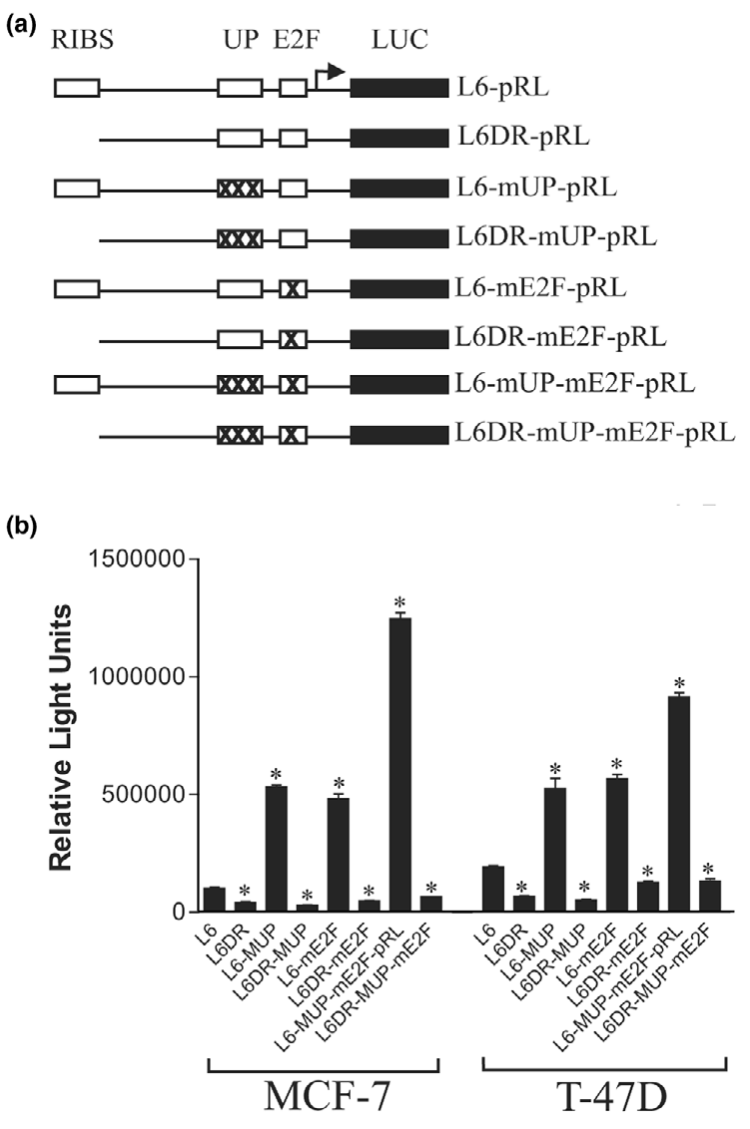
Analysis of the effect of mutation of the UP site on *BRCA1 *proximal promoter activity. **(a) **Schematic representation of the *BRCA1 *renilla-luciferase reporter plasmids: mutations were introduced in the context of both the full-length proximal promoter (L6-pRL) and a promoter construct from which the RIBS site had been deleted (L6DR-pRL). Point mutations were made in the UP site (L6-mUP-pRL, L6DR-mUP-pRL) and the downstream E2F binding site (L6-mE2F-pRL, L6DR-mE2F-pRL), or both sites in combination (L6-mUP-mE2F-pRL, L6DR-mUP-mE2F). See (b) for sequences. **(b) **Transfection assays for the effects of the various mutants on promoter activity. MCF-7 and T-47D cells were cotransfected with 225 ng of one of the *BRCA1 *promoter constructs described above and 25 ng of the internal control vector (CMV-Luc). Both renilla and firefly luciferase values were measured using a dual luciferase assay. The data presented is a representative experiment of mean values of triplicates ± the standard deviation of the relative light units of the pRL reporter constructs normalized to the luciferase activity of the internal control vector. Independent experiments were performed a minimum of three times. Statistical significance was determined using a *t*-test and significant results (p = 0.05) are indicated by asterisks and are in relation to the L6 vector.

To assess the functional significance of this site for *BRCA1 *promoter activity, a reporter construct was created with mutations of all six of the nucleotides identified as being critical for the binding of the complex to the UP site. These mutations were made in the context of the *BRCA1 *L6 promoter, which extends from nucleotide -208 to +27 and which we have previously determined to have optimal promoter activity in human breast tumour lines (Figure 2a). This point mutant, referred to as L6-mUP, was transfected into MCF-7 and T-47D cell lines. In both cell lines the L6-mUP construct exhibited a three- to five-fold increase in promoter activity compared to the wild-type L6 promoter (Figure 2b). This suggests that the UP site functions as a repressor element in these lines.

### The UP and E2F sites form a composite repressor element

**Figure 3:**
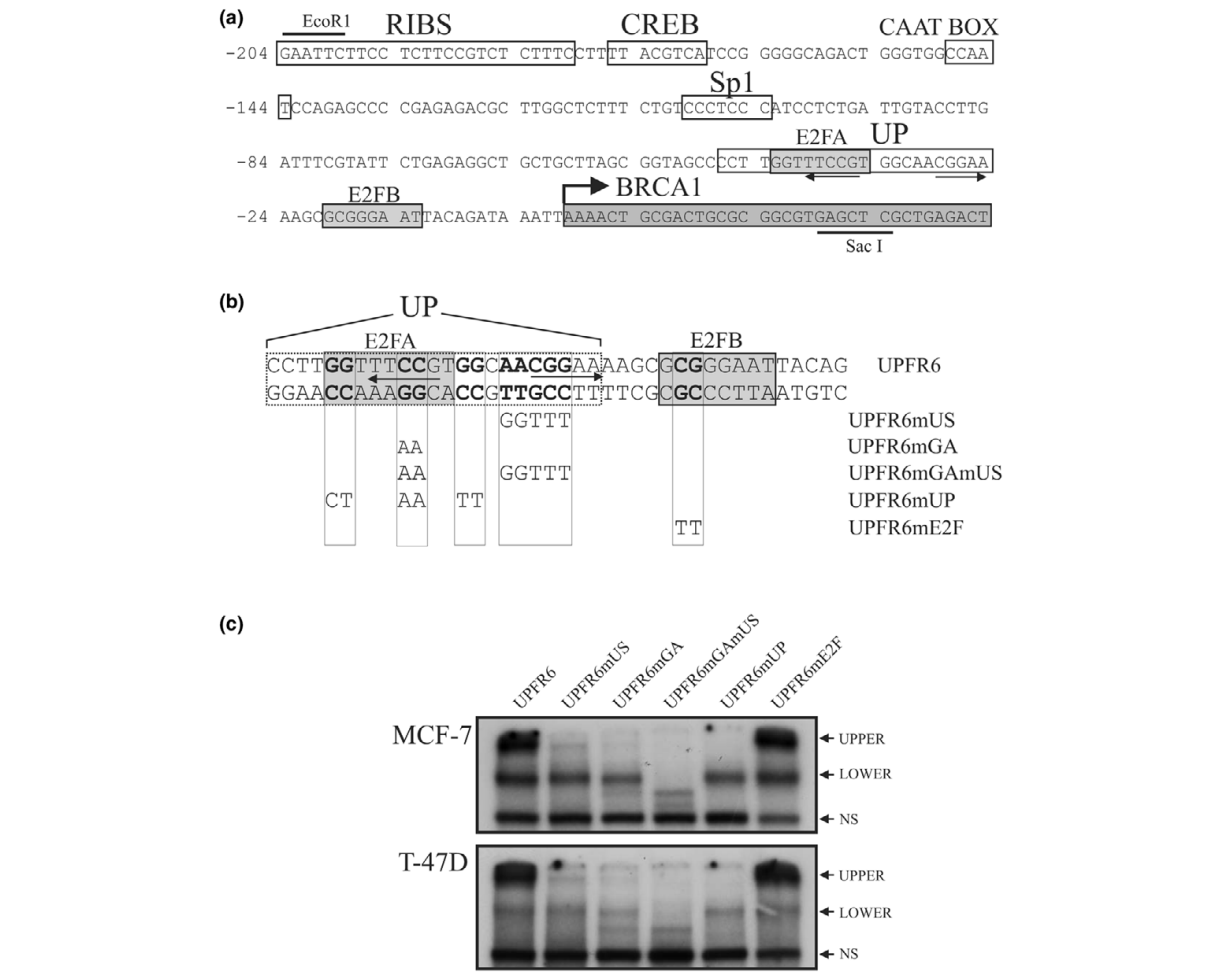
Effects of mutations in the UP site on the binding of endogenous nuclear proteins. **(a) **The sequence of the *BRCA1 *proximal promoter is shown with previously characterized sites boxed. E2F sites characterized by Bindra and Glazer [27] (E2FA and E2FB) are shown by light grey boxes while the *BRCA1 *first exon is shown by the darker box and by the arrow showing the transcriptional start site. The arrows indicate the potential *ets*/GABP binding sites. **(b) **The sequence of the UPFR6 probe is indicated, which encompasses both the UP and E2F sites (dotted and grey box marked E2FB). Arrows identify putative GABP alpha/beta binding sites. The sequences of the various mutant probes are indicated along with their names. **(c) **The ^32^P-labelled UPFR6 probes described in (b) were incubated in binding reactions with 5 μg of nuclear extract from the MCF-7 and T-47D cell lines and bandshift assays were performed. Only the DNA/protein complexes are shown, with the Upper, Lower and non-specific (NS) bands indicated as in Figure 1.

The presence of an E2F site in the *BRCA1 *promoter has been previously reported and was thought to act as an element mediating E2F-6 repression [24]. This element is immediately downstream of the UP site (Figures 2a and 3a). Mutation of the E2F site in the context of the L6 promoter (L6-mE2F) resulted in increased expression in both the MCF-7 and T-47D cell lines, with the activity being comparable to that of the L6-mUP construct (Figure 2b). Mutation of both sites together produces higher expression than the single mutants, with the effect being approximately additive (Figure 2b, L6-mUP-mE2F). These two elements appear to be part of a composite repressor element where mutation of either site results in loss of repression of the promoter.

### Derepression of a *BRCA1 *promoter construct is dependent upon the RIBS site

We have previously identified a GABP alpha/beta site, which we refer to as the RIBS element, upstream of the UP site in the *BRCA1 *proximal promoter [16]. The RIBS site is required for optimal promoter activity and is part of the minimal bidirectional transcription element that is involved in the expression of both the *BRCA1 *gene and the divergently expressed *NBR2 *gene [15]. Deletion of this element in the context of the L6 promoter construct decreases its expression significantly and has a similar effect on the activity of the UP and E2F single-site mutants in both the MCF-7 and T-47D cell lines (Figure 2b, L6DR). In MCF-7 cells the construct with both the RIBS deletion and the UP mutation (L6DR-mUP) has comparable activity to the L6DR mutant, which lacks only the RIBS site. This suggests that the derepression resulting from mutation of the UP site may be dependent on the function of the RIBS element. A similar effect is observed with the double RIBS and E2F mutant (L6DR-mE2F) and with the triple RIBS, UP and E2F mutant (L6DR-mUP-mE2F). These results are generally comparable in T-47D cells.

### Multiple complexes assemble on the UP site

The possible presence of a composite complex with repressor activity occurring on the UP and E2F sites led us to investigate the effect of additional mutations in the context of the UPFR6 probe, which includes both the UP and E2F sites (Figure 3b). A five base-pair region downstream of the original UP mutations was mutated (UPFR6mUS) and when tested in bandshift assays with nuclear extracts from both MCF-7 and T-47D cells resulted in the loss of the upper complex (UPPER) seen with the wild-type probe but retained the lower (LOWER) and non-specific (NS) complexes (Figure 3c). Similarly, the original UP mutation (UPFR6mUP) or a two base-pair mutation (UPFR6mGA) corresponding to the sequence of the middle UP mutation also resulted in the loss of the upper complex. The double GA and US mutant (UPFR6mGAmUS) resulted in loss of binding to the middle complex but appears to produce an even lower novel complex. These results suggest that the upper complex is composed of at least two proteins, one binding to the sequence defined by the GA mutant and another factor binding to the US region. Disruption of binding of either of these proteins results in an intermediate complex, and there is the suggestion that a third protein may bind as the double mutant still interacts with a faster migrating complex. This factor could be interacting with the E2F site, although mutation of this element alone does not result in a change of protein complexes. Given the large size of the upper complex, the effect of the E2F mutation might not be visible, and may be revealed only when the other sites are mutated.

### GABP alpha/beta binds directly to the UP site

**Figure 4:**
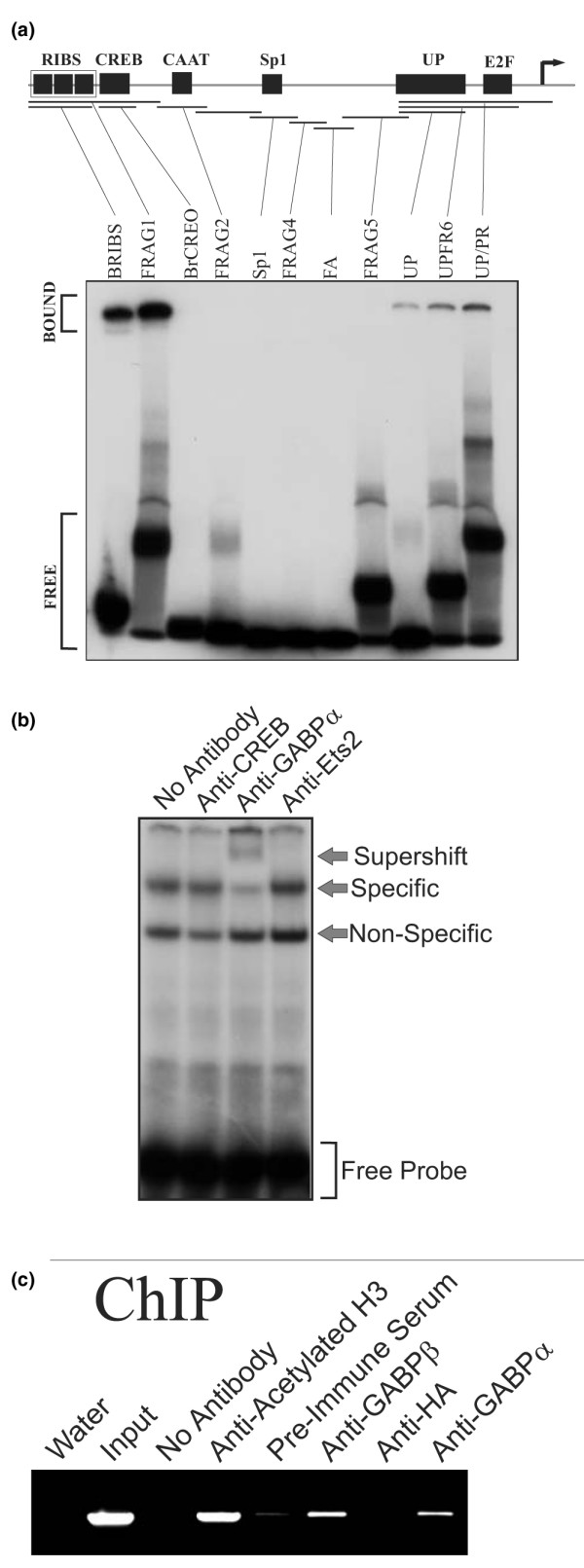
Binding of recombinant, nuclear extract derived and endogenous GABP alpha/beta to the *BRCA1 *promoter. **(a) **A bandshift scanning assay of the *BRCA1 *promoter for GABP complex binding sites was preformed using recombinant GABP alpha/beta. Double-stranded DNA probes spanning putative protein binding sites were designed for the entire length of the *BRCA1 *proximal promoter and are indicated on the schematic (top). Binding reactions were performed with recombinant human GABP alpha and beta proteins and each of the ^32^P-labelled probes as indicated by the vertical lines. Samples were run on a 6% nondenaturing acrylamide gel and visualized by autoradiography. The locations of the free and bound probe are indicated. **(b) **Supershift assays with the UPFR6 probe were performed with 5 μg of MCF-7 nuclear extract and an antibody directed against CREB, GABP alpha or Ets-2. Complexes are as indicated. **(c) **ChIP assays were preformed using chromatin isolated from MCF-7 cells with antibodies against acetylated histone H3, GABP beta, GABP alpha and haemagglutinin (HA). PCR products obtained using *BRCA1*-specific primers and the immunoprecipitation products are shown.

Inspection of the sequence of the UP element reveals several different potential recognition elements for previously characterized transcription factors. These include two general *ets *factor binding sites (GGAA) that are also preferential binding sites for the *ets *factor GABP alpha/beta (CGGAA), one on each of the coding and non-coding strands (Figure 3a,b, arrows) that correspond to both the GA and US binding sites for nuclear proteins. GABP alpha/beta sites are often found as direct repeats, as the protein can form heterotetramers on two such elements [25], but in the UP site they are inverted repeats. In order to determine if the UP element, or any other element in the promoter, could bind GABP alpha/beta, a series of overlapping double-stranded oligonucleotide bandshift probes were generated spanning the promoter. This comprehensive approach, which we refer to as bandshift scanning, allows for the specific identification of all binding elements within the proximal promoter. We then used recombinant GABP alpha/beta dimers in bandshift assays with these probes. As expected, a strong complex was seen with the BRIBS probe as well as with a slightly larger overlapping probe, FRAG1, as both contain the previously characterized GABP alpha/beta binding RIBS element [16] (Figure 4a). In addition, a complex was seen with three other probes, UP, UPFR6 and UP/PR. The minimal UP element appears to be sufficient to bind recombinant GABP alpha/beta. The ability of this site to bind GABP alpha/beta was confirmed by the use of a supershift assay with an antibody to the GABP alpha subunit. A distinct supershift is seen when nuclear extracts from MCF-7 (Figure 4b) or T-47D cells (data not shown) were used.

To confirm the *in vivo *occupancy of the promoter by GABP alpha/beta, we also carried out ChIP assays using antibodies directed against GABP and a PCR assay targeted to the human BRCA1 promoter. MCF-7 chromatin was precipitated with various controls, including no antibody (Figure 4c, No Antibody), pre-immune serum from the rabbit used to raise antibodies against the GABP beta subunit (Figure 4c, Pre-Immune Serum), and an affinity purified anti-heamagglutinin tag antibody (Figure 4c, Anti-HA). All of these negative controls gave no or minimal PCR product. A general positive control using antibodies against acetylated histone H3 (Figure 4c, Anti-Acetylated H3) gave a robust product as expected. Antibodies against both GABP beta (Figure 4c, Anti-GABPβ) and GABP alpha (Figure 4c, Anti-GABPα) also gave a positive signal, confirming the presence of GABP alpha/beta on the *BRCA1 *promoter. Due to the lack of spacial resolution inherent in the ChIP assay it is impossible to determine if the binding of GABP alpha/beta corresponds to interaction with the RIBS, UP or both sites but indicates that it is able to interact with this promoter.

**Figure 5:**
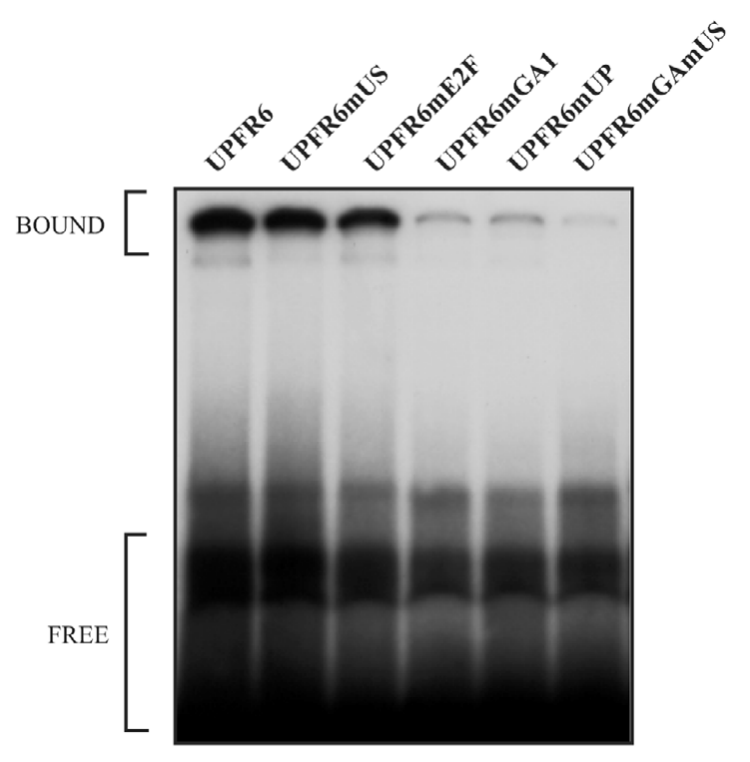
Effects of mutations in the UP site on the binding of recombinant GABP alpha/beta. Bandshift reactions were performed with recombinant human GABP alpha and beta proteins and ^32^P-labelled wild-type (UPFR6) and mutant UPFR6 site probes, as described in Figure 4a with the probes described in Figure 3b. Complexes are as indicated.

The mutant UPFR6 sites used to characterize the binding of nuclear proteins to the UP site were also assayed for their ability to bind recombinant GABP alpha/beta. The UPFR6mUP probe, which alters the upstream putative GABP element (Figure 5, UPFR6mUP), as well as a probe containing a two base-pair mutation of this upstream GABP element alone (Figure 5, UPFR6mGA) resulted in loss of GABP alpha/beta binding. Mutation of the downstream putative GABP site (Figure 5, UPFR6mUS) had no effect on recombinant GABP alpha/beta binding, while the double mutant (Figure 5, UPFR6mGAmUS) also resulted in the loss of GABP alpha/beta binding. It thus appears that the upstream CGGAA sequence in the UP site is necessary for the binding of GABP alpha/beta, while the downstream CGGAA sequence is not.

### Both the GA and US mutants of the UP site result in *BRCA1 *promoter activation

**Figure 6:**
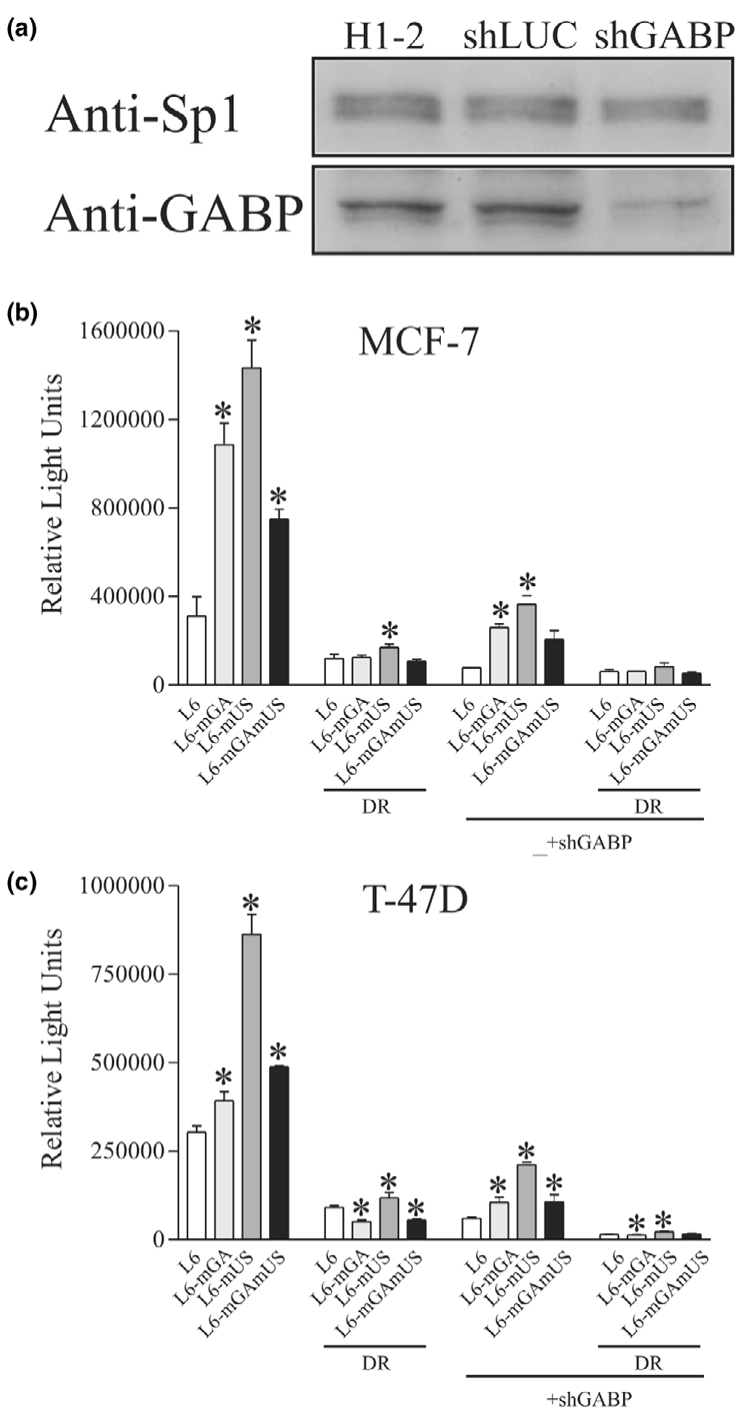
Fine mapping the UP site using point mutants. **(a) **An shRNA vector knocks down GABP alpha. HeLa cells were transfected with an empty shRNA vector (H1–2) or vectors directed against the luciferase gene (shLUC) or the GABP alpha gene (shGABP) and protein lysates collected and analysed by western blot. Control antibodies against Sp1 (Anti-Sp1) or antibodies recognizing GABP alpha (Anti-GABP) were used to detect these proteins. **(b,c) **The mGA and mUS point mutations alone (L6-mGA, L6-mUS) and in combination (L6-mGAmUS) were incorporated into the L6 or L6 with the RIBS site deleted (DR) promoters. These were then cotransfected into MCF-7 (b) or T47D (c) cell lines with the empty H1–2 vector or with the GABP alpha shRNA vector (+shGABP). Cells were harvested 48 hours post-transfection and analysed for luciferase activity. The data presented are the mean values ± standard deviation of a representative experiment performed in triplicate and normalized as described in Figure 2. Statistical significance was determined using a *t*-test and significant results (p = 0.05) are indicated by asterisks and are in relation to the L6 or L6DR vector in each group.

The GA mutant clearly affects GABP alpha/beta binding and while the US mutant does not, it does affect the formation of the upper complex associated with the UP site. Both mutations, alone and in concert, were introduced into the L6 reporter construct and assayed for activity in MCF-7 and T-47D cells. In MCF-7 cells all three mutant constructs resulted in greater promoter activity compared to the wild-type L6 construct (Figure 6b). Interestingly, the double GA/US mutant was less active than either of the single mutants alone. This may indicate that mutation of either site results in derepression of the UP element, thereby unmasking the effect of other proteins that then act as activators. Mutation of both sites therefore mediates derepression, but also abolishes some of this additional activation. This suggests then that GABP bound to the GA site, and some as yet unidentified protein associated with the US site, can both independently function to activate the promoter once repression has been lifted. Similar results were observed in T-47D cells, although the effect of the GA mutation was much less (Figure 6c). This finding implies that the factor associated with the US site in MCF-7 cells is absent or unable to mediate transactivation in this line. As with the UP and E2F mutants, removal of the RIBS site results in an overall loss of promoter activity (Figure 6b,c, deleted RIBS (DR)).

### shRNA knockdown of GABP alpha/beta inhibits *BRCA1 *promoter activity through the RIBS and UP sites

In order to assess the effect of endogenous GABP alpha/beta levels on *BRCA1 *promoter activity, an shRNA vector was created that targeted the alpha subunit of GABP alpha/beta. This construct was able to efficiently down-regulate GABP alpha/beta protein levels by 60% to 80% when transfected into HeLa cells (Figure 6a) or MCF-7 cells (data not shown). The activity of the L6 promoter is dramatically reduced by the cotransfection of the shRNA vector in both MCF-7 and T-47D cell lines, indicating that GABP alpha/beta is an important regulator of the *BRCA1 *promoter in these lines (Figure 6b,c, +shGABP). The activities of the constructs containing the GA and US UP site mutations, both of which result in loss of repression of the promoter, are also greatly decreased by the GABP alpha shRNA in both the MCF-7 and T-47D lines, although complete loss of activity is not achieved. This may be the result of incomplete knock-down of the GABP complex. Removal of the RIBS site from these constructs, however, results in further decreases in activity as well as the abrogation of all mutation-specific activity (Figure 6b,c, deleted RIBS (DR) +shGABP).

### RIBS and UP multimer sites act as GABP alpha/beta-dependent activator elements

**Figure 7:**
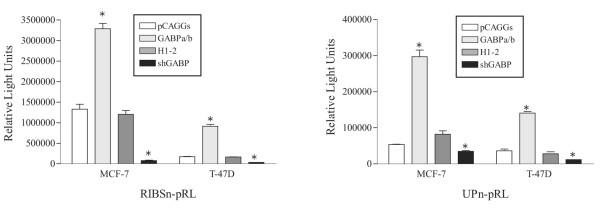
Cotransfection of the GABP alpha/beta expression vectors and the GABP alpha shRNA with RIBS and UP site multimer reporter constructs. MCF-7 and T-47D cells were transiently transfected with 125 ng of GF-TATA-renilla-luciferase reporter containing oligomerised RIBS or UP sites (RIBSn-pRL, UPn-pRL), 25 ng of internal control vector (CMV-Luc), and either 25 ng each of GABP alpha and beta expression constructs (light grey bars), or 50 ng of their empty control vector (pCAGGs; white bars), or 50 ng of GABP alpha shRNA (black bars) or its empty control vector (H1–2; dark grey bars). Cells were harvested 48 hours post-transfection and analysed for luciferase activity. The data presented are the mean values ± standard deviation of a representative experiment performed in triplicate and normalized as described in Figure 2. Statistical significance was determined using a *t*-test and significant results (p = 0.05) are indicated by asterisks and are in relation to the empty vector control for each condition (pCAGGS or H1–2).

To confirm the activities of the individual GABP alpha/beta binding sites we cloned multimers of the RIBS and UP sites upstream of a TATA box-containing minimal promoter [16]. The RIBS multimer was transactivated by cotransfection of the GABP alpha/beta expression vectors in MCF-7 and T-47D lines (Figure 7). The shRNA vector dramatically decreased promoter activity in both cell lines. The UP multimer behaved in a similar manner to the RIBS multimer reporter, with GABP alpha/beta cotransfection increasing activity in both lines. The shRNA vector reduced the activity of the UP multimer in MCF-7 and T-47D lines but the degree of this decrease was not as great as for the RIBS element. In isolation, both the RIBS and UP elements appear to act as GABP alpha/beta dependent activator elements in MCF-7 and T-47D cell lines.

## Discussion

### A composite repressor element is present in the *BRCA1 *promoter

The structure and regulation of the *BRCA1 *promoter has been of particular interest due to the association of decreased *BRCA1 *gene transcription with the development of sporadic breast cancer [12]. We have identified a new element in the *BRCA1 *promoter that functions as a transcriptional repressor. The sequence of this element is well conserved between human and mouse promoters (16/18 bases), emphasizing its importance for regulation of this gene. Inactivation of the UP element using point mutations results in a three- to five-fold increase in expression in two different human breast cancer cell lines. We also determined that a previously identified E2F binding site [26] immediately downstream of the UP site is also able to act as a repressor in breast tumour cells. The repressor element in the *BRCA1 *promoter appears to extend from the UP through to the E2F element, though these appear to have independent functions as mutation of both sites is additive. The E2F site was originally identified as a potential mediator of increased *BRCA1 *expression in response to the induction of growth, likely mediated through E2F-1 [26]. Subsequently, E2F-6 activity was associated with repression of the *BRCA1 *promoter using an shRNA approach and its binding was thought to occur in a reciprocal manner with E2F-1 to regulate the promoter [24]. Bindra and Glazer [27] independently characterized two E2F sites within the proximal *BRCA1 *promoter, their E2FB site (Figure 3a,b) being coincident with the previously identified downstream E2F site, and a second E2F recognition element within the UP site that overlaps with our GA element. These sites appear to bind both E2F1 and E2F4 and may be regulated in turn by interaction with p130/p107. In agreement with our results, mutation of either site was shown to increase promoter activity; however, the E2FA site (the UP site) was neither necessary nor sufficient to bind E2F proteins as judged by DNA capture assays [27]. Overall, these results emphasize that a composite repressor element encompasses both the UP and E2F sites, though the question of the composition and partners of the E2F proteins involved remains complex.

### The role of GABP alpha/beta in promoter regulation

We have previously identified GABP alpha/beta as a critical regulator of the *BRCA1 *promoter acting through the RIBS element [16] and in this paper we have characterized a second GABP alpha/beta site within the UP element. The RIBS element is crucial in that it is required for basal *BRCA1 *promoter activity as well as being essential for the derepression of the *BRCA1 *promoter resulting from mutations in the UP site (Figure 6, wild-type RIBS verses DR mutants). In contrast to its interaction with the RIBS element, where it acts solely as an activator, the binding of GABP alpha/beta to the UP element appears to also have a repressor function as mutation of the GABP alpha/beta recognition element leads to loss of UP mediated repression. However, when the UP site is taken out of the context of the promoter and multimerised, GABP alpha/beta was shown to activate this site. This is in keeping with previous observations that GABP alpha/beta can act as either an activator or repressor depending on the specific context of the promoter it interacts with [19,28]. The function of the GABP complex is thought to be influenced by the composition of the heterodimerisation partner of the DNA-binding alpha subunit. Differential splicing of the GABP beta gene generates the gamma subunit, which interacts with GABP alpha but does not allow for tetramerisation [25,29]. The GABP alpha/gamma subunit is thus thought to function as a repressor, acting in opposition to the GABP alpha/beta complex. We have not been able, however, to detect the gamma subunit protein product in these breast cancer cell lines (data not shown), suggesting this is not the mechanism by which the GABP complex regulates the activity of the UP site.

**Figure 8:**
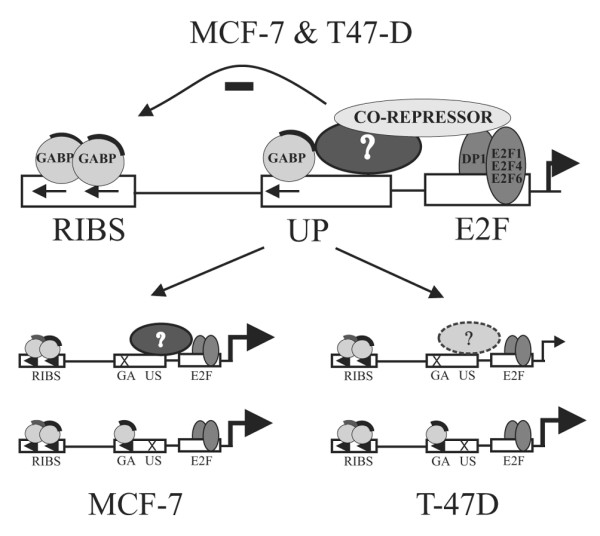
Model of GABP alpha/beta interaction with the UP site and its effect on *BRCA1 *promoter activity. Schematic representations of the regulation of the *BRCA1 *promoter. In both MCF-7 and T-47D cells (upper diagram) the RIBS and UP sites, respectively, are shown to bind GABP alpha/beta (shaded circles represent the DNA binding alpha subunit, with the beta subunit indicated by the dark line) while the E2F site has previously been suggested to bind E2F1, E2F4 and E2F6. The UP site also binds another unknown protein (oval) and together the UP and E2F sites are able to recruit an unknown co-repressor. The GA mutant of the UP site (x) disrupts binding of GABP alpha/beta while the US mutant affects the binding of the other unidentified factor. In T-47D cells this other factor does not appear to be functional (dashed oval). See text for explanation.

Both the GA and US mutants of the UP site result in the loss of the large molecular weight complex (Figure 3) and induce similar levels of derepression (Figure 6). This may be due to cooperative interactions between these proteins in which loss of either protein results in decreased complex formation and failure to form a repressor complex (Figure 8). The observation in MCF-7 cells that the GA/US double mutant promoter construct has lower activity than either of the single mutants (Figure 6), suggests that once unmasked by loss of the repressor complex, the factors bound to these sites are individually able to mediate activation of the *BRCA1 *promoter. In T-47D cells the US mutant exhibits an increase in activity similar to what was seen in the MCF-7 cell line. In contrast, the level of activity seen with the GA mutant is comparable to that of the GA/US mutant in this line and both constructs are significantly less active than in the MCF-7 line. As the complexes obtained by Electrophoretic Mobility Shift Assay (EMSA) using the various UP site probes with either MCF-7 or T-47D nuclear extracts are similar (Figure 3c), it would appear that the factor associated with the US site in T-47D cells is present on the promoter, but is either inactive or missing a functional co-activator (Figure 8).

### Implications for promoter function

A recent report has identified 53BP1 as a positive regulator of the *BRCA1 *promoter that acts through sequences in the UP element [30]. 53BP1 contains a BRCA1 carboxy-terminal domain, localizes to sites of double-strand breaks, and activates the Ataxia Telangiectasia Mutant (ATM) pathway [31]. A small interfering RNA directed against 53BP1 represses *BRCA1 *expression, while an expression vector activates the promoter. It is suggested that induction of *BRCA1 *expression by DNA damage could be mediated by 53BP1. However, a mutant within the UP site, which abrogates 53BP1 binding based on bandshift and ChIP assays, still responds to 53BP1 overexpression, suggesting its effect may be indirect. These results were obtained primarily in U2OS cells, which are a human osteosarcoma derived cell line, rather than in breast cells. 53BP1 does not bind to DNA in a sequence specific manner, suggesting that its effect is likely mediated through its recruitment by other transcription factors. It is possible that 53BP1 may modulate the repressor and or activator functions of the UP site during periods of DNA damage to bring about an increase in BRCA1 levels. However, its role in constitutive expression of BRCA1 is not clear.

The E2F family is directly involved in mediating cell cycle regulation and it is known that *BRCA1 *expression increases in response to growth [26,32]. Similarly, GABP alpha/beta has been implicated in the cell cycle regulation of genes such as *Skp2 *[33] and indeed appears to regulate a growth mediated pathway distinct from that of the D-type cyclins [34]. By incorporating both of these factors into a composite regulatory element, the UP/E2F site may be critical for integrating signals coming from different growth activated pathways that determine the nature and level of *BRCA1 *expression.

The *BRCA1 *promoter is part of a bidirectional transcription unit that also directs expression of the *NBR2 *gene [15]. The UP element is outside of the minimal bidirectional transcription unit that is able to direct transcription in both directions. Its location near the start site for *BRCA1 *expression may mean that it plays a role exclusively in regulating *BRCA1 *expression, while elements that regulate *NBR2*/*BRCA1 *directionality actually lie farther up and downstream of this region [35].

## Conclusion

The identification of a repressor element in the *BRCA1 *promoter brings a new level of complexity to the regulation of *BRCA1 *expression. Given the critical role that decreased *BRCA1 *expression has in the development of sporadic breast cancer, the study of mechanisms that can down-regulate this key tumour suppressor are of particular importance. The elements characterized here may play a normal role in the integration of a variety of signals, including two different growth related pathways, and it is possible that loss of the ability to derepress the *BRCA1 *promoter during critical periods may contribute to breast transformation.

## Abbreviations

ChIP = chromatin immunoprecipitation; shRNA = small hairpin RNA.

## Competing interests

The authors declare that they have no competing interests.
